# Perioperative Anaphylaxis to Chlorhexidine during Surgery and Septoplasty

**DOI:** 10.1155/2017/9605804

**Published:** 2017-03-19

**Authors:** Ana Paula Teixeira de Abreu, Leonardo Ramos Ribeiro de Oliveira, Ana Flavia Teixeira de Abreu, Evandro Ribeiro de Oliveira, Michele Santos de Melo Ireno, Fernando Monteiro Aarestrup, Matheus Fonseca Aarestrup, Paula Fonseca Aarestrup

**Affiliations:** ^1^Hospital Evandro Ribeiro, Rua Catarina de Castro, 75 Morro da Glória, 36036-060 Juiz de Fora, MG, Brazil; ^2^Serviço de Alergia e Imunologia Clínica Hospital Maternidade Therezinha de Jesus e Faculdade de Ciências Médicas e da Saúde de Juiz de Fora (SUPREMA), Alameda Salvaterra, 200 Salvaterra, 36033-003 Juiz de Fora, MG, Brazil; ^3^Instituto de Ensino Superior Presidente Tancredo de Almeida Neves (IPTAN), Avenida Leite de Castro, 1101 Fábricas, 36301-182 São João del Rei, MG, Brazil; ^4^Escola de Medicina e Cirurgia da Universidade Federal do Estado do Rio de Janeiro (UNIRIO), Silva Ramos, 32 Tijuca, 20270-330 Rio de Janeiro, RJ, Brazil

## Abstract

Chlorhexidine is an antiseptic and disinfectant used in surgical and clinical practice since 1954 and is available in aqueous or alcoholic solutions 0.5%–4.0% and has a broad-spectrum activity. Despite their widespread use, allergic reactions with chlorhexidine are rarely reported. We describe a case of anaphylaxis with chlorhexidine during a septoplasty, turbinectomy, and maxillary sinusectomy. The patient presented with periorbital edema, hives, hypotension, and wheezing. Immediately after the diagnosis of anaphylaxis promethazine, hydrocortisone, and epinephrine were administered with immediate clinical improvement. This case highlights the importance of assessing whether there is a previous clinical history of hypersensitivity to chlorhexidine in patients who will undergo surgical procedures.

## 1. Introduction

Chlorhexidine is an antiseptic and disinfectant found in many products, such as toothpastes, plasters, ointments, suppositories, cleansing fluids, and sterilizers, among other products used daily [1, 2]. It has been used in clinical practice as an antiseptic since 1954 [3] and is available in aqueous or 0.5%–4.0% alcoholic solutions. Chlorhexidine has broad-spectrum activity against gram-positive and gram-negative bacteria, facultative anaerobes, yeast, and HIV [4–8].

Among other applications, chlorhexidine is also used in combination with mupirocin to eliminate methicillin-resistant* Staphylococcus aureus* from the nasal vestibule [9]. Moreover, during septoplasty, standard solutions, such as povidone iodine or chlorhexidine, are used for skin preparation [10]. The use of chlorhexidine during surgical procedures is not specified in medical records because it is extensively used as an antiseptic solution; thus, the incidence of allergic reactions may be underestimated [5, 9].

There are two types of hypersensitivity reactions to chlorhexidine [1, 2, 5, 6]. The immediate reactions are characterized by the onset of symptoms occurring within 30 minutes after exposure to the causative agent and are associated with the production of specific IgE class antibodies that bind to receptors on the surface of mast cells and basophils promoting the release of various inflammatory mediators. This type of reaction presents clinically as urticaria and angioedema and may progress to anaphylaxis compromising the cardiovascular and respiratory systems and leading to death. Allergy to chlorhexidine may also manifest as a delayed hypersensitivity reaction that occurs between 48 and 72 hours after exposure and usually presents as contact dermatitis. These late reactions are mediated by T lymphocytes and do not present a risk of serious systemic reactions.

The increased exposure rate to chlorhexidine might have contributed to sensitization and the appearance of IgE-mediated anaphylaxis, which is sometimes fatal or near-fatal, particularly in the context of surgical procedures [5]. Anaphylaxis results from the intense release of proinflammatory mediators from mast cells and basophils. Histamine and tryptase are the major mediators involved in the anaphylaxis pathogenesis. Prior contact with the agent is required for the development of this type of immediate hypersensitivity reaction. Target organs include the skin, mucous membranes, and the respiratory, cardiovascular, and gastrointestinal systems [11–15].

Skin prick test and intradermal test with 0.5% and 0.0002% chlorhexidine gluconate, respectively, may be used for diagnostic purposes of allergic reactions [5]. Careful evaluation of the clinical history and appropriate allergy testing are the main tools for discovering the causative agent of anaphylaxis. In this article we present a case of chlorhexidine-induced anaphylaxis during a surgical procedure where adequate diagnostic investigation was performed allowing the identification of the allergen that triggered the immediate hypersensitivity reaction.

## 2. Case Presentation

A 25-year-old woman presented with anaphylactic reaction during a septoplasty, turbinectomy, and maxillary sinusectomy. Midazolam hydrochloride, fentanyl, and propofol were used for anesthesia induction. Chlorhexidine was used for mucosal and skin antisepsis. Approximately after 30 minutes the patient had periorbital edema, hives, hypotension wheezing, and decreased oxygen saturation. Promethazine 0.1 mg was administered intramuscularly, hydrocortisone 500 mg was administered intravenously, and 0.5 ml of epinephrine 1 : 1,000 was administered intramuscularly in the* vastus lateralis* muscle of the tight, resulting in immediate clinical improvement.

In order to identify the cause of anaphylaxis, the patient was referred to the Allergy and Immunology Department of the Hospital of the School of Medical and Health Sciences (SUPREMA), Minas Gerais State, Brazil. The prick test and intradermal test were carried out with drugs used during the surgery, namely, chlorhexidine, lidocaine, cephalothin, acetylcysteine, chloramphenicol, cephalosporin, midazolam, fentanyl, and propofol. The prick test was also performed with latex. Both allergic tests were positive for chlorhexidine and negative for the other substances. After a detailed anamnesis, the patient reported prior lip swelling when mouthwash containing chlorhexidine gluconate was used.

Informed consent was obtained from the patient and all work was conducted in accordance with the principles outlined in the Declaration of Helsinki (1964).

## 3. Discussion

The use of chlorhexidine appears to have increased, since studies demonstrated that this product is more effective in preventing infections than povidone iodine. Moreover, daily domestic use might cause allergic sensitization in the general population [5, 8, 15]. Allergic reactions to chlorhexidine can be classified into two types. Immediate hypersensitivity reactions are mediated by IgE class antibodies and occur within 30 minutes of exposure to the causative agent. In these cases there is a risk of an anaphylactic reaction occurring which, if not diagnosed and treated properly, can lead to death. Late hypersensitivity reactions occurring 48 to 72 hours after exposure to chlorhexidine may also be seen clinically manifesting as allergic contact dermatitis and not presenting a risk of serious systemic reactions [8, 9, 15].

The real incidence of allergy to chlorhexidine is unknown. Therefore, these reactions might be more prevalent, and cases might have been ignored, due to a lack of suspicion of chlorhexidine as a causative agent [5, 9, 15]. Despite the wide spectrum of use of chlorhexidine for medical purposes, chlorhexidine anaphylaxis is considered relatively rare [9, 12, 15].

Complications during surgery with general anesthesia can have multiple causes, including allergic reactions. The estimated overall rate of anaphylaxis is 1 per 10,000–20,000 anesthetic procedures [14]. Anaphylaxis is a severe systemic allergic reaction that affects two or more systems, including the skin, respiratory, cardiovascular, and gastrointestinal systems [15]. Since chlorhexidine has become widely used in hospitals and private healthcare [5, 9–11, 15, 16], it has increasingly been reported to be a potential allergenic agent. Diagnostic investigation of the cause of perioperative anaphylaxis should be carefully performed by a medical specialist in Allergy and Immunology. All possible allergic substances used during the surgery should be carefully investigated.

Surgeons and anesthetists should be aware of chlorhexidine as a potential allergen associated with anaphylaxis during surgical procedures. In the present case, the patient reported prior lip swelling after using mouthwash containing chlorhexidine. Therefore during the preoperative consultation all patients should be investigated as to the previous clinical history of allergy to chlorhexidine. Finally, we hope that this study may contribute to alerting health professionals about the potential of chlorhexidine to cause severe allergic reactions in previously sensitized patients.
